# More epigenetic hits than meets the eye: microRNAs and genes associated with the tumorigenesis of retinoblastoma

**DOI:** 10.3389/fgene.2012.00284

**Published:** 2012-12-07

**Authors:** Adriana H. O. Reis, Fernando R. Vargas, Bernardo Lemos

**Affiliations:** ^1^Genetics Program, Instituto Nacional de CâncerRio de Janeiro, Brazil; ^2^Genetics and Molecular Biology Department, Universidade Federal do Estado doRio de Janeiro (UNIRIO), Brazil; ^3^Molecular and Integrative Physiological Sciences Program, Department of Environmental Health, Harvard School of Public HealthBoston, MA, USA

**Keywords:** retinoblastoma, methylation, two-hit hypothesis, imprinting, Rb1, tumor suppressor, risk assessment, childhood cancer

## Abstract

Retinoblastoma (RB), a childhood neoplasia of the retinoblasts, can occur unilaterally or bilaterally, with one or multiple foci per eye. RB is associated with somatic loss of function of both alleles of the tumor suppressor gene *RB1*. Hereditary forms emerge due to germline loss of function mutations in *RB1* alleles. RB has long been the prototypic “model” cancer ever since Knudson's “two-hit” hypothesis. However, a simple two-hit model for RB is challenged by an increasing number of studies documenting additional hits that contribute to RB development. Here we review the genetics and epigenetics of RB with a focus on the role of small non-coding RNAs (microRNAs) and on novel findings indicating the relevance of DNA methylation in the development and prognosis of this neoplasia. Studies point to an elaborated landscape of genetic and epigenetic complexity, in which a number of events and pahtways play crucial roles in the origin and prognosis of RB. These include roles for microRNAs, inprinted loci, and parent-of-origin contributions to *RB1* regulation and RB progression. This complexity is also manifested in the structure of the *RB1* locus itself: it includes numerous repetitive DNA segments and retrotransposon insertion elements, some of which are actively transcribed from the *RB1* locus. Altogether, we conclude that *RB1* loss of function represents the tip of an iceberg of events that determine RB development, progression, severity, and disease risk. Comprehensive assessment of personalized RB risk will require genetic and epigenetic evaluations beyond *RB1* protein coding sequences.

## Introduction

Retinoblastoma (RB), a childhood neoplasia of the retinoblasts, accounts for 2–4% of all childhood malignancies. Its prevalence is around 1:15,000–1:20,000 (MacCarthy et al., 2006; Broaddus et al., 2009), and can occur unilaterally or bilaterally, with one or multiple foci per eye. RB can occur sporadically (~60% of cases) or be inherited (~40% of cases) in an autosomal dominant fashion (Cavenee et al., 1983; Lohmann and Gallie, 2004; Balmer et al., 2006). Both forms of this malignancy, hereditary and sporadic, are associated with somatic loss of function of both alleles of the tumor suppressor gene *RB1*. Hereditary forms are also associated with germline loss of function of one of the *RB1* alleles (Knudson, 1971; Cavenee et al., 1983; Friend et al., 1986). The RB1 protein (pRb) is known to have crucial roles in cell cycle control and differentiation, being involved in the G1/S transition via repression of the E2F transcription factor essential for S phase initiation (Giacinti and Giordano, 2006).

It might be surprising that loss of function of *RB1*, a crucial component in the control of the cell cycle, has its highest impact on a small population of cells, namely the cone precursors in the developing retina. However, the highest expression of pRb in the retina was indeed observed in cone precursors, suggesting a stronger antiproliferative role in these cells (Xu et al., 2009). It was also demonstrated that MDM2 and NMYC are highly expressed in maturing human cone precursors. MDM2 can abrogate E2F- and ARF-mediated apoptotic responses by inhibiting p53. Using lentiviral short harpin RNA vectors to decrease MDM2 or NMYC expression, Xu et al. (2009) have demonstrated impairment in cell proliferation, induction of apoptosis, and increased proportion of G0/G1 cells. These findings are consistent with a cone precursor origin of RB and a crucial role for MDM2 in the development and maintenance of the tumor.

RB has long been the prototypic “model” cancer, ever since Knudson's comparison of the age of diagnosis of bilateral versus unilateral patients led to his “two-hit” hypothesis for the initiation of cancer (Knudson, 1971). However, these events, although apparently sufficient to initiate a tumor, are modified by the presence of numerous additional genetic changes in RB tumors. Evidence from developing retinal cells indicates that two mutational events are not enough for malignant transformation (Chen et al., 2004), and an increasing number of studies have suggested that other hits are associated with RB development. Indeed, several genes and pathways have shown functional dysregulation and misexpression in RB, and pointed to surprising ways in which the expression of *RB1* can be modulated. Here we review the genetics and epigenetics of RB with focus on the role of small non-coding RNAs (microRNAs) and molecular mechanisms involved in the development and prognosis of this neoplasia.

## MicroRNAs and retinoblastoma

**Figure 1:**
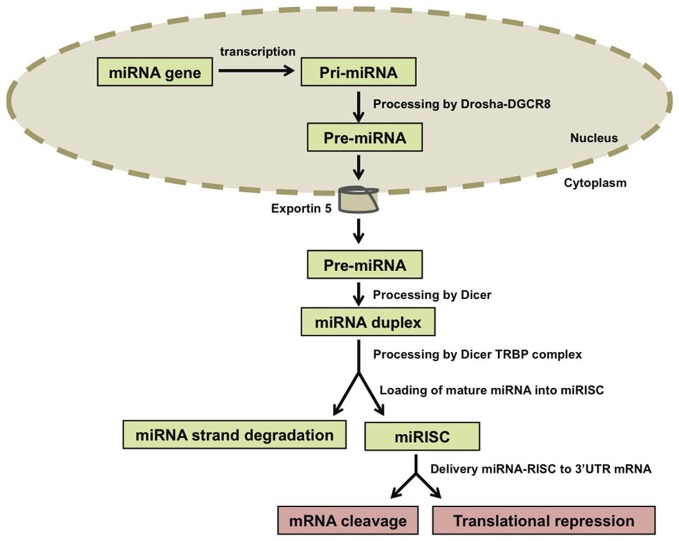
**Biogenesis of microRNAs (miRNAs).** miRNA genes are transcribed and the resulting primary transcripts (pri-miRNAs) are polyadenylated at the 3′ end and capped at the 5′ end. Pri-miRNA molecules are recognized by the Drosha-DGCR8 complex and trimmed in a precursor-miRNA (pre-miRNA) that is transported to the cytoplasm by Exportin-5. In the cytoplasm, Dicer processes the pre-miRNAs and one miRNA duplex is released from each pre-miRNA. The two strands of the duplex are separated from each other by the Dicer-TRBP complex and one of the strands (called mature miRNA) is incorporated into the RNA-induced silencing complex (RISC) that will target specific mRNAs in a sequence-dependent manner. The other strand, which is not incorporated into RISC, is called the miRNA strand and is degraded.

MicroRNAs (miRNAs) encode small non-coding RNAs that have been recognized as a large gene class present in most organisms. MicroRNAs are molecules 18–25 nucleotides long that are produced through two cleavage steps. These steps are accomplished by two distinct complexes between an RNA III enzyme and double-strand RNA-binding domain proteins (DROSHA-DGCR8 and DICER-TRBP). Mature miRNAs are incorporated into miRNA-protein complexes that bind to the 3′ untranslated region (3′UTR) of mRNA targets (He and Hannon, 2004; Zhao et al., 2009). These complexes are referred to as micro-ribonucleoproteins (miRNPs) or miRNA-induced silencing complexes (miRISCs). The best characterized components of miRNPs are proteins of the Argonaute family (Peters and Meister, 2007; Filipowicz et al., 2008). The binding of miRNA-protein complexes to mRNAs species inhibits translation or destabilize the target transcript resulting in the downregulation of the protein product encoded by the respective mRNA (Figure 1).

MicroRNAs are expressed in a tissue-specific and developmental stage-specific manner, providing an additional layer of complexity in the regulation of gene expression. Through the parallel modulation of the expression of target transcripts, miRNAs can coordinately alter many different signaling pathways and cellular processes such as proliferation, differentiation, or apoptosis. Accordingly, the control of critical targets by miRNAs has multiple implications in cancer (He and Hannon, 2004; Bueno and Malumbres, 2011).

At least 10% of the human genome is regulated in a cell cycle-dependent manner (Iyer et al., 1999) and miRNAs are known to be functionally integrated into many crucial cell cycle control pathways. Many miRNAs are antiproliferative, and this function may be mediated by control of different mitogenic pathways, including the routes that lead to activation of CDKs. A few miRNAs induce proliferation by targeting CDK inhibitors or members of the pRB family (Bueno and Malumbres, 2011).

The discovery of differentially expressed miRNAs in human RB is recent and has been replicated with a variety of techniques. These include real time qPCR, microarray, and deep sequencing. Altogether, several several miRNAs are emerging as candidate components of oncogene and tumor suppressor networks in RB. Table 1 shows some differentially expressed miRNAs in RB tumor when compared to normal retina.

**Table 1:** **microRNAs that are differentially expressed in human retinoblastoma**.

**microRNAs**	**References/Methods**
let-7e; miR-513; miR-518c; miR-129; miR-198; miR-320; miR-373; miR-492; miR-494; miR-498; miR-503	Zhao et al., 2009/A
let7a; let-7f; miR-2; miR-7; miR-9; miR-16; miR-17a, miR-20a; miR-25; miR-26a; miR-30b; miR-30d; miR-92a; miR-93a; miR-96; miR-99b; miR-101; miR-103; miR-106b; miR-124; miR-143; miR-148b; miR-181a; miR-183; miR-216a; miR-217; miR-378; miR-1246	Conkrite et al., 2011/A
let-7a; let-7b; let-7c; miR-10a; miR-10b; miR-20a; miR-21; miR-28; miR-29b; miR-30a-3p; miR-30b; miR-30c; miR-30d; miR-99a; miR-99b; miR-100; miR-103; miR-107; miR-124a; miR-125a; miR-125b; miR-133a; miR-136; miR-141; miR-145; miR-146a; miR-155; miR-181a, miR-181b; miR-182; miR-183; miR-190; miR-191; miR-206; miR-210; miR-222; miR-301; miR-302a; miR-302b; miR-320; miR-330; miR-335; miR-342; miR-368; miR-373; miR-380-5p; miR-382; miR-423; miR-433; miR-451; miR-452; miR-491	Li et al., 2012/A
miR-34a	Dalgard et al., 2009/B; C
let-7c; let-7i; let-7g; miR-10a; miR-10b; miR-28-5p; miR-29a; miR-29b; miR-29c; miR-34a; miR-34b; miR-34c-5p; miR-96; miR-99a; miR-100; miR-124; miR-125b; miR-130a; miR-132; miR-135b; miR-137; miR-142-3p; miR-142-5p; miR-149; miR-181a; miR-182; miR-183; miR-193a-3p; miR-193b; miR-199a-3p; miR-214; miR-224; miR-338-3p; miR-363; miR-374a; miR-375; miR-376a; miR-505	Jo et al., 2011/A

*A, microarray analysis; B, semiquantitative RT-PCR; C, real-time qPCR*.

The majority of differentially expressed miRNAs in RB were also described in association with other cancers. miR-373, for instance, was identified in a functional screen for miRNAs that promote cell migration *in vitro* (Huang et al., 2008), and its prometastatic potential has been validated in tumor transplantation experiments using breast cancer cells. Furthermore, miR-373 has been identified as a potential oncogene in testicular germ cell tumors (Voorhoeve et al., 2006). However, it has been proposed that the prometastatic and oncogenic properties of this miRNA are due to the regulation of distinct sets of genes (Voorhoeve et al., 2006; Ventura and Jacks, 2009). Zhao et al. (2009) showed that, in RB tumors, miR-373 expression level was increased compared to normal retina, suggesting a role in tumor pathway.

### Tumor suppresor miRNAs in retinoblastoma

Members of the let-7 family are among the most widely studied tumor suppressor miRNAs. The first member of this family was discovered in the worm *C. elegans*, where it induces cell cycle exit and terminal differentiation of a specific cell type at the transition from larval to adult stages (Reinhart et al., 2000). Coherent with a role in inhibiting tumor development in humans, meager expression of multiple members of the let-7 family is correlated with poor prognosis in lung cancer (Yanaihara et al., 2006). Functionally, let-7 represses members of the Ras family of oncogenes (Johnson et al., 2005) as well as HMGA2 and c-Myc oncogenes (Lee and Dutta, 2007; Mayr et al., 2007; Sampson et al., 2007). In a recent study, reduced expression levels of let-7 were observed in RB (Mu et al., 2010). Additionally, an inverse correlation between let-7 down-regulation and HMGA2 overexpression was documented (Mu et al., 2010).

The miR-34 family of miRNAs, located in 1p36, is also associated with tumor suppressor functions. Studies have demonstrated that p53 transcriptionally activates the miR-34 family (Chang et al., 2007; He et al., 2007; Raver-Shapira et al., 2007). Additionally, p53-mediated transcriptional activation of miR-34 family members contributes to p53-dependent tumor suppression through cell cycle arrest and activation of apoptosis (Chang et al., 2007; He et al., 2007; Raver-Shapira et al., 2007; Tarasov et al., 2007). Loss or silencing of miR-34a has been identified in several human cancers, including brain, breast, colorectal, lung, pancreatic, and prostate; these observations make miR-34a an attractive microRNA for therapeutic development (Dalgard et al., 2009). In the two commonly used RB cell lines (Y79 and Weri-Rb1). miR-34a shows variable expression levels, and it has been suggested that knockdown of miR-34a by anti-miR molecules may result in increased RB cell proliferation and chemotherapeutic resistance (Dalgard et al., 2009). These authors also observed additional RB cell growth inhibition when topotecan was used combined with miR-34a. This result is in agreement with previous findings of similar growth inhibition in a combined treatment using topotecan and the p53 activator nutlin-3 (Laurie et al., 2006). Altogether, the evidence supports the notion of a tumor suppressor role for miR-34a in the normal retina.

### Oncogenic miRNAs in retinoblastoma

Several oncogenic miRNAs have also been identified. The miR17~92 cluster (OncomiR-1) has been studied in a broad range of cancers. miR-17~92 cluster belongs to a highly conserved family of polycistronic miRNA genes, whose two other members are the miR-106a~363 cluster and the miR-106b~25 cluster (Ventura et al., 2008; Sage and Ventura, 2011). Studies in mice showed that these three clusters share partially overlapping targets and functions, with miR-17~92 being essential for mammalian development. Nevertheless, the relative contribution of the various miRNAs encoded by the three clusters is poorly understood (Ota et al., 2004; Olive et al., 2010).

Recurrent focal amplifications of the miR-17~92 locus were reported in a significant fraction of diffuse large B-cell lymphomas (Ota et al., 2004; Olive et al., 2010), and overexpression of the six miRNAs encoded by this cluster was observed in a wide variety of solid and liquid tumors (Ventura et al., 2008). Mir-17~92 carries out pleiotropic functions during both normal development and malignant transformation. It acts to promote proliferation, inhibit differentiation, increase angiogenesis, and sustain cell survival (Olive et al., 2010). In RB, miR-17~92 acts via proliferation control to promote tumorigenesis, at least in part via direct suppression of key cell cycle inhibitors such as p21^CIP1^ and p57^KIP2^ (Conkrite et al., 2011). The experiments of Conkrite et al. (2011) suggested that miR-17~92 synergizes with loss of Rb family members to promote RB. miR-17~92 genomic amplifications were observed in murine RB, and high expression of miR-17~92 was observed in human RB. However, while miR-17~92 was dispensable for mouse retinal development, miR-17~92 overexpression, together with deletion of pRb and p107, led to rapid emergence of RB with frequent metastasis to the brain. Furthermore, these authors found that deletion of Rb family members led to compensatory up-regulation of the cyclin-dependent kinase inhibitor p21Cip1. MiR-17~92 overexpression counteracted p21Cip1 up-regulation, promoted proliferation, and drove RB formation (Conkrite et al., 2011). Another study (Nittner et al., 2012) investigated whether the survival function of miR-17~92 was applicable to human tumors that harbor *RB1* mutations. In order to address this question, components of the miR-17~92 cluster were inactivated using miRNA inhibitors in the human RB cell lines RBL15, WERI-RB1, and Y79. Results indicated that miR-17~92 inactivation suppresses RB formation in mice, and co-silencing of miR-17/20a and p53 cooperatively decreases the viability of human RB cells (Nittner et al., 2012). The authors suggested that RB cells might be addicted to high levels of miR-17~20a expression as a result of a synthetic lethal interaction with both p53 and RB pathways. Together with additional recently reported data (Olive et al., 2010), these findings identify miR-17 and miR-20a (from miR17~92 cluster) as putative therapeutic targets for the selective prevention and/or treatment of RB (Nittner et al., 2012).

Studies investigating miRNA expression patterns in different RB cell lines indicated that the differentially expressed miRNAs in two RB cell lines show different impact on tumor growth: SNUOT-Rb1 cell line shows adherent and more rapid growth, while Y79 cells display non-adherent and slower growth (Jo et al., 2011). The authors suggested that many targets of the overexpressed miRNAs in each cell line have specific biological functions, which would impact RB progression, like cell adhesion, cell cycle, cell death, and cell division. Interestingly, while some differentially expressed miRNAs were known as oncogene miRNAs, other miRNAs were regarded as tumor suppressor miRNAs in other tumors (Jo et al., 2011).

**Figure 2:**
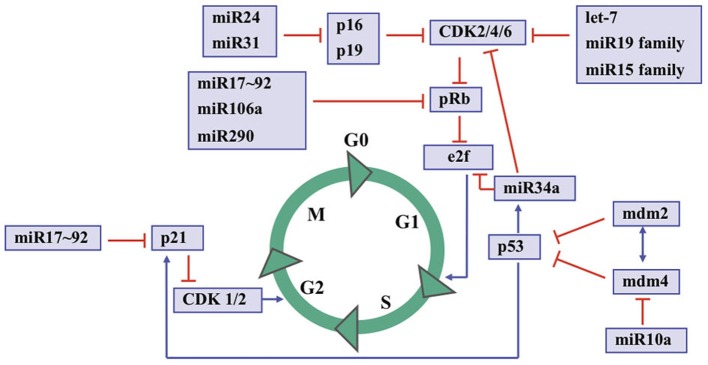
**An overview to cell cycle control by microRNAs and some key proteins.** S indicates the S-phase; M indicates the mitosis; G1 and G2 indicate transition phases of the cell cycle whereas G0 indicates quiescent cells.

Altogether, the data presented by studies in miRNAs and RB not only provide novel insights into the pathogenesis of this neoplasia, but also may have therapeutic potential for decreasing oncogenic activity, increasing tumor suppressive function, and/or promoting differentiation in a very large group of cancer patients. Figure 2 shows an overview of the cell cycle control by microRNAs and some proteins. However, further studies on *in vitro* and *in vivo* activities of specific miRNAs and their role in normal cell types are required for their reliable use as a biomarker for risk assessment and as a therapeutic target.

## Protein coding genes associated with retinoblastoma

### Differential gene expression profile in retinoblastoma

Recently a great number of studies have documented the differential gene expression profile of RB compared to normal retina. Ganguly and Shields (2010), identified 1116 genes with increased expression and 837 genes with decreased expression in RB tumor tissue compared to matched normal retinal tissue from six individuals. Functional categories of the cognate genes with the greatest statistical support were cell cycle (309 genes), cell death (437 genes), DNA replication, recombination and repair (270 genes), cellular growth and proliferation (464 genes), and cellular assembly and organization (110 genes). The list included differentially expressed retinal cone cell-specific markers, indicating the predominance of cone cells in RB, and supporting the notion that the latter group of cells may be the cells of origin for RB (Xu et al., 2009; Ganguly and Shields, 2010).

Ganguly and Shields (2010) suggested that genes differentially expressed in RB belong mainly to DNA damage response pathways, including *BRCA1*, *BRCA2*, *ATM*, *ATR*, *E2F*, and *CHK1* genes. In addition, the authors suggested the involvement of additional and novel pathways. These include pathways involved in aryl hydrocarbon receptor (AHR) signaling, polo-like kinase and mitosis, and purine metabolism. Molecules AHR, CHK1, and polo-like kinases are of particular interest because there are several currently available drugs that target these molecules (Ganguly and Shields, 2010). Also, AHR forms a complex and synergizes with pRb to repress E2F-dependent transcription and cell cycle progression (Puga et al., 2000). This finding suggests an additional mechanism through which environmental signals can function to activate pRb. Accordingly, one of the critical functions of AHR appears to be that it acts as an environmental sensor that, in the presence of environmental toxicants, binds to pRb, and signals cell cycle arrest (Puga et al., 2000).

Chakraborty et al. (2007) used microarrays to analyze gene expression profile of RB, and observed 1004 upregulated, and 481 downregulated genes compared to retina samples from normal controls. Clusters of differentially expressed genes were identified on chromosomes 1, 16, and 17. Based on the expression profile, the authors hypothesized that the PI3K/AKT/mTOR (insulin signaling) pathway might be dysregulated in RB. This was supported by semiquantitative RT-PCR analysis of *PIK3CA*, *AKT1*, *FRAP1*, and *RPS6KB1* genes in RB samples, suggesting that the potential therapeutic use of known inhibitors of this pathway (Chakraborty et al., 2007).

An investigation of RB-related genes with shortest path in a protein–protein interaction network identified a total of 527 genes and ranked them by their betweenness (Li et al., 2012). The top 119 genes with betweenness greater than 100, and *p*-value <0.05 were further analyzed owing to their potential importance in the regulatory network of RB. Intriguingly, the authors found genes with no previous association with RB, or that have been only poorly characterized. These include *CDC25C*, *NOTCH1*, *CDC6*, *PLK3*, and *VAV1*. *CDC25C* is an important phosphatase in cell cycle control, particularly for entry into mitosis, and the increase in CDC25C has been documented in multiple cancers with poor prognosis (Iguchi et al., 2007). Notch signaling plays an essential role in the processes of embryogenesis and cellular differentiation. It has been reported that elevated Notch signaling and epigenetic silencing of *RB1* in Drosophila can lead to tumorigenesis (Axelson, 2006). CDC6 is an essential regulator of DNA replication in eukaryotic cells. Its main function is the assembly of pre-replication complexes at origins of replication during the G1 phase of the cell division cycle. Moreover, CDC6 also plays critical roles in the activation and maintenance of G1-S checkpoint, as an essential regulator of initiation of DNA replication. Elevated levels of CDC6 was observed in many human cancer cells (Li et al., 2012). *PLK3* is a member of the Polo-like kinases family that becomes phosphorylated following DNA damage or mitotic spindle disruption (Bahassi El et al., 2002). Some studies have shown that *PLK1* expression is elevated in many cancers including non-small cell lung cancer, head and neck cancer, colorectal cancer, and others. *PLK1* gene and protein expression have been regarded as new prognostic markers for many types of malignancies, as well as a potential target for cancer therapy (Takai et al., 2005). Proto-oncogene *VAV1* is a member of the Dbl family of guanine nucleotide exchange factors (GEF) for the Rho family of GTP binding proteins. It has been shown that *VAV1* is ectopically expressed in a large number of cancers, including pancreatic adenocarcinoma, neuroblastoma, melanoma, and B-cell chronic lymphocytic leukemia (Li et al., 2012).

### Candidate oncogenes and tumor suppressor genes in retinoblastoma

RB represents one of the rare cancers in which the initiating genetic lesion (*RB1* loss of function) is known, and provides an excellent system in which to identify new genes, which may, like *RB1*, have broad importance for cancer development (Corson and Gallie, 2007). Evidence for additional events (termed M3 to Mn in keeping with Knudson's nomenclature) in RB, along with epigenetic modification are being documented at a fast pace. Studies performing karyotype analyses, comparative genomic hybridization (CGH) and microarray-based CGH (aCGH) showed recurrent Mn events composed by chromosomal gains and losses (Corson and Gallie, 2007). These recurrent events identify candidate oncogenes and tumor suppressor genes that might play important roles in RB. Accordingly, gain of 1q, 2p, 6p, and 13q, and loss of 16q were the most frequently observed chromosomal aberrations. Potential candidate genes included *KIF14*, *MDM4*, *MYCN*, *E2F3*, *DEK*, *CDH11*; aberrant methylation of *MGMT*, *RASSF1A*, *CASP8*, and *MLH1* genes was also identified (Corson and Gallie, 2007).

Although the development and/or progression of RB is not typically associated with *TP53* gene mutations (Huang, 1999), recent studies have suggested that dysregulation or inactivation of the p53 pathway might also be relevant in RB (Laurie et al., 2006; de Oliveira Reis et al., 2012). Accordingly, amplification of two oncogenes associated with p53 regulation, *MDM2*, and *MDM4*, as well as polymorphisms in these genes have been associated with RB development and/or survival (Laurie et al., 2006; de Oliveira Reis et al., 2012). Polymorphisms rs2279744 T>G in *MDM2* and rs116197192 G>A in *MDM4*, were investigated in RB patients and compared to controls. The MDM2 rs2279744G allele was significantly more frequent in controls, pointing to a possibly protective effect on RB development. However, the survival of patients who carried a constitutional *RB1* mutation was significantly lower with rs2279744TG or GG than with rs2279744TT genotypes. Presence of the rs2279744G allele and a constitutional *RB1* mutation was six-fold more frequent in the 0–12 month age group than other age groups at onset of symptoms. MDM4 rs116197192G allele was present at a significantly higher frequency in patients suggesting that this allele might increase the risk of developing RB. Results indicated that *MDM2* and *MDM4* polymorphisms might influence development and/or survival in RB (de Oliveira Reis et al., 2012). Laurie et al. (2006) showed that, during retinogenesis, the tumor surveillance pathway mediated by ARF, MDM2, MDM4, and p53 is activated after loss of pRb. RB1-deficient retinoblasts undergo p53-mediated apoptosis and exit the cell cycle. Subsequently, amplification of *MDM4* and increased expression of MDM4 are strongly selected for during tumor progression, as a mechanism to suppress p53 response in RB1-deficient retinal cells. Altogether, the data provided evidence that p53 pathway is inactivated in RB, and that this cancer does not necessarily originate from intrinsically death-resistant cells as previously thought.

**Figure 3:**
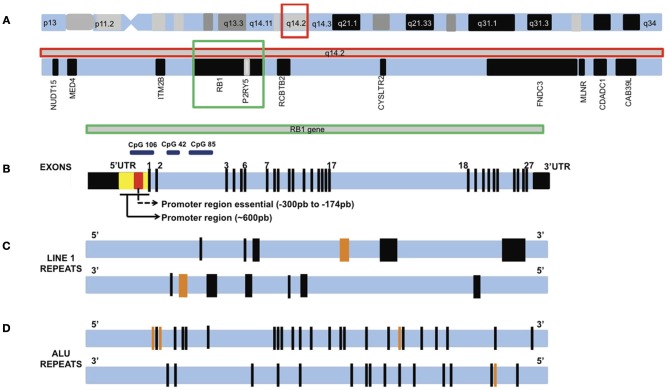
**Chromosome 13 and the *RB1* gene (not drawn to scale). (A)** Chromosome 13 and the q14.2 band highlighted in a red square. Inside q14.2 band are *RB1* gene (highlighted in a green square) and flanking genes. **(B)** *RB1* gene showing exons 1–27. Black boxes are untranslated regions (UTR) 5′ and 3′. Two promoter regions investigated in methylation studies are shown inside the 5′UTR. A large number of MSP studies investigated the essential promoter region, while the MLPA studies investigated part of these two regions. These regions concentrate a large number of CpG island, like CpG 106, CpG 42, and CpG 85. **(C)** A pair of horizontal lines showing the location of LINE1 repeats in *RB1* gene oriented in the sense direction (top line) and antisense direction (bottom line). Each black bar represents one LINE1, orange bars represent two LINE1 repeats. The bar thickness is proportional to the size of the sequence. **(D)** A pair of horizontal lines showing the location of ALU repeats in *RB1* gene oriented in the sense direction (top line) and antisense direction (bottom line). Each black bar represents one ALU repeat, orange bars represent two ALU repeats.

Studies using other neoplasias have also contributed to identify chromosomal regions that contain genes relevant for *RB1* regulation. Lee et al. (2007) used human bladder cancer as a model system to identify clonal genetic hits associated with growth advantage, tracking the evolution of bladder cancer from intraurothelial precursor lesions. Six putative chromosomal regions critical for clonal expansion of intraurothelial neoplasia and development of bladder cancer were identified. In particular, Lee et al. (2007) identified a 1.34-Mb segment around the *RB1* gene associated with the initial expansion of the neoplasia. This segment contained additional candidate genes (Figure 3A) that may contribute to cancer progression; Interesting targets include two neighbor genes flanking *RB1*, namely *ITM2B* and *RCBTB2*, as well as *P2RY5*, which is located inside *RB1*. Indeed, Lee et al. (2007) reported that *ITM2B* and *P2RY5* modulated cell survival and were silenced by methylation or point mutations, respectively. Their functional loss may contribute to the growth advantage of cancer derivatives. The authors also showed that homozygous inactivation of *P2RY5* was antecedent to the loss of *RB1* during tumor development, and that nucleotide substitutions in *P2RY5* increased cancer risk. Hence, epigenetic alterations that target not only *RB1* but also its flanking genes might significantly contribute to RB emergence and progression.

## Methylation in retinoblastoma

Greger et al. (1989) were the first to show that CpG 106, which overlaps the *RB1* promoter and exon E1, is methylated in some RB tumors. This was one of the first hints that promoter methylation of a tumor suppressor gene plays a role in tumorigenesis. Subsequently, Sakai et al. (1991) investigated methylation marks at the 5′ end of the *RB1* gene in DNA purified from 56 primary RBs. The study found five tumors with evidence of hypermethylation (Sakai et al., 1991). Later, Ohtani-Fujita et al. (1993) found that *in vitro* methylation of the *RB1* promoter in a reporter gene construct reduced gene expression by 92% (Ohtani-Fujita et al., 1993). Furthermore, they have identified two transcription factors that did not bind to *RB1* when their recognition sequences were methylated (Ohtani-Fujita et al., 1993). These studies as well as that of Toguchida et al. (1993), identified high densities of CpG sequences in a region encompassing exon 1 and in a second region in intron 2 of the *RB1* gene. It was hypothesized that these regions might be associated with loss of function of the *RB1* gene due to allele specific hypermethylation (Toguchida et al., 1993). Since then, many studies have documented *RB1* promoter methylation (Ohtani-Fujita et al., 1997; Richter et al., 2003; Corson and Gallie, 2007; Livide et al., 2012), and have shown an epigenetic component of RB tumorigenesis.

Kanber et al. (2009) have identified novel imprinted loci by genome wide CpG methylation analysis of DNA from the blood of patients with methylation disorders. A 1.2 kb CpG island with parent-of-origin-specific methylation was identified inside intron 2 of the *RB1* gene—the segment is methylated on the maternal chromosome 13 and unmethylated on the paternal chromosome 13. This CpG island (CpG 85) serves as a promoter for an alternative transcript of *RB1*, which is expressed from the unmethylated paternal chromosome only. The first exon of this alternative transcript is E2B, which is spliced onto exon E3. This feature distinguishes CpG 85 from two other CpG islands associated with the *RB1* gene: CpG 42, which is located a few kilobases upstream of CpG 85, and CpG 106, which overlaps the *RB1* promoter and exon E1. CpG 42 is biallelically methylated, whereas, CpG 106 is biallelically unmethylated (Buiting et al., 2010). Figure 3B shows the location of CpGs 106, 42, and 85 on *RB1* gene. If the alternative transcript (*RB1*) were expressed in addition to and independently of the regular paternal transcript, then the total level of paternal transcripts should be higher than that of the maternal transcripts. However, analysis of parent-of-origin-dependent expression of *RB1* transcripts revealed a 3-fold excess of maternal *RB1* mRNA (Kanber et al., 2009). In mice, which do not have the intronic CpG island, no parent-of-origin-specific expression imbalance was found, indicating that skewed allelic expression of the human pRb is linked to the differentially methylated CpG 85. This notion was further substantiated by the finding that demethylation of CpG 85 in lymphoblastoid cell lines by 5-aza-20-deoxycytidine treatment resulted in reduced skewing of the allelic *RB1* transcripts, which is to be expected because after loss of CpG 85 methylation the maternal allele resembles the paternal allele (Kanber et al., 2009). Authors suggested that allele-specific methylation of CpG 85 affects expression of pRb, probably by transcriptional interference (a mechanism in which the transcription of one gene has a suppressive influence on the transcription of another gene). It is plausible that differential penetrance and variation of age at diagnosis, which have been observed in patients with hereditary and non-hereditary RB, respectively, are a consequence of imprinted expression. The direction of the imprint imposed on *RB1* gene is the same as that of the maternally expressed *CDKN1C* gene, which operates upstream of *RB1* (Kanber et al., 2009).

Interestingly, analyses by Kanber et al. (2009) suggested that CpG 85 is part of a 4.5 kb region with a high sequence identity (87%) to exon 4 of KIAA0649, a four-exon gene in segment 9q34.3. The four small (~300 bp) CpG islands present in exon 4 of KIAA0649 appear to have evolved into two CpG islands (CpG 85 and CpG 42) following integration into the *RB1* gene (retrotransposition event). Specifically, CpG 85, which spans 1.2 kb, corresponds to the small islands CpG 19 and CpG 17 at the KIAA0649 locus, which only contain 229 bp and 209 bp, respectively (Kanber et al., 2009). Other transposition events are important for the stability and expression of the *RB1* gene. One case is a 799-bp deletion in the intron 2 of *RB1*. It was identified in a group of brain tumors and probably emerges due to a homologous recombination between two Alu repeats (Rothberg et al., 1997). Figures 3C,D show LINE1 and ALU repeats distribution in the *RB1* gene.

The methylation status of other genes has also been studied in RB. Analysis of *p16INK4A* promoter methylation in RB showed hypermethylation in most patients with *p16INK4A* downregulation, and in their parents (Indovina et al., 2010; Saxena and Kaur, 2011). The finding that *p16INK4A* was downregulated both in RB patients and their parents suggests that this alteration could be a novel heritable susceptibility marker to RB. The observation that *p16INK4A* downregulation seems to be due to its promoter hypermethylation opens the way for the development of new preventive and therapeutic strategies using demethylating agents (Indovina et al., 2010; Saxena and Kaur, 2011).

Liu et al. (2009) investigated the methylation status of *RASSF1A* or *DAPK* promoter in 28 RB tissues, normal retinal tissues from five donor cadaver eyes, nine peripheral blood samples of RB patients, and normal-peripheral blood samples from five healthy volunteers matched on age and sex by methylation-specific PCR (MSP). The authors found that the percentage of *RASSF1A* hypermethylation in RB tissues (60.7%) was statistically higher (*p* < 0.01) than in the normal retinal tissue (0%) and in the adjacent non-neoplastic retinal tissue (17.9%). The hypermethylation percentage in unilateral RB group was also higher than in the bilateral RB group. On the other hand, *DAPK* promoter hypermethylation was not found in any RB tumor or peripheral blood sample. *RASSF1A* is a tumor suppressor whose inactivation is implicated in the development of many human cancers (Liu et al., 2009). Its methylation status and role in RB was demonstrated previously by Harada et al. (2002), who found *RASSF1A* promoter methylation in 59% of RB tumors (Harada et al., 2002).

Recently, Zhang et al. (2012) identified the *SYK* gene as a potentially important oncogene in RB. This gene is the fifth most significant gene identified by their analysis and the only kinase gene that is upregulated. *SYK* regulates immunomodulatory signaling, it is expressed in the haematopoietic system, and has been implicated in several haematologic malignancies (Chen et al., 2008; Feldman et al., 2008; Hahn et al., 2009; Young et al., 2009; Zhang et al., 2012). Interestingly, this protein has no known function in the developing visual system, with retinal progenitor cells and retinal neurons expressing little or no SYK (Zhang et al., 2012). Moreover, no recurrent genetic lesions in *SYK* had been identified by WGS or SNP array analysis to suggest that this gene drives RB tumorigenesis. Epigenetic profiles, however, showed profound changes in *SYK* relative to that observed in normal retinoblasts. *SYK* is required for tumor cell survival, and inhibition of SYK with a small-molecule inhibitor caused the degradation of MCL1 and caspase-mediated cell death in RB cells in culture and *in vivo* (Zhang et al., 2012).

Livide et al. (2012) used Methylation-specific Multiplex Ligation-dependent Probe Amplification (MS-MLPA) to interrogate epigenetic and copy number variation in 12 paraffin embedded RB tissue samples. The authors identified promoter hypermethylation in seven genes (proportion of hypermethylated tumors is shown in parenthesis): *MSH6* (50%), *CD44* (42%), *PAX5* (42%), *GATA5* (25%), *TP53* (8%), *VHL* (8%), and *GSTP1* (8%). Also, the study replicated the previously reported hypermethylated genes: MGMT (58%), *RB1* (17%), and CDKN2 (8%). These genes belong to key pathways, including DNA repair, pRB and p53 signaling, transcriptional regulation, protein degradation, cell–cell interaction, cellular adhesion, and migration. In the same study, a total of 29 copy number changes (19 duplications and 10 deletions) were identified. Interestingly, they have found deletions of the following oncosuppressor genes that might contribute to drive RB tumorigenesis: *TP53*, *CDH13*, *GATA5*, *CHFR*, *TP73*, and *IGSF4* (Livide et al., 2012). Figure 3B shows a graphical presentation of *RB1* gene with the main promoter regions and CpG islands investigated in methylation studies.

Finally, parent-of-origin effects have been reported in human phenotypes associated with mutations in *RB1*. These include parental effects on the differential penetrance and age of onset in RB, and an excess of first somatic mutations on paternal alleles in sporadic osteosarcoma (Toguchida et al., 1989; Klutz et al., 2002; Richter et al., 2003; Schuler et al., 2005). Altogether, the evidence points to a greater role for epigenetic alterations in RB. Nevertheless, recent advances in genomics and epigenomics have made it possible to study RB in novel ways. Microarray and sequencing technologies in association with multiple complementary technologies, including CGH, serial analysis of gene expression (SAGE), chromatin immunoprecipitation (ChIP), proteomics, and deep sequencing have been used to study all aspects of cancer biology. Their judicious application might help uncover the molecular mechanism of cancer development and have an impact on diagnosis, prognosis, drug responses, and new therapeutic approaches in cancer. Combining the results of these multidisciplinary approaches will contribute to a better biological understanding of RB and an improved clinical management of RB patients. *RB1* loss has long been recognized as the causative genetic alteration underlying RB but it is increasingly evident that other genetic and epigenetic alterations are also required for the tumor to develop. Recent findings highlight how comprehensive genetic and epigenetic analyses of tumors can be integrated to shed light on the mechanisms underlying the progression of RB following *RB1* inactivation.

## Conclusions

RB represents one of the rare cancers in which the initiating genetic lesion (*RB1* loss of function) is known. Yet, the genetic and epigenetic complexity of RB is widely appreciated, and evidenced by the intricate network of cellular and epigenetic components that modulate *RB1* expression. These include transcription factors and chromatin-modifying proteins that target the *RB1* locus. This complexity is also manifested in the structure of the *RB1* locus itself: it includes numerous repetitive DNA segments and retrotransposon insertion elements, some of which are actively transcribed from the *RB1* locus. Evidence indicates that some of these repetitive elements are targeted in spurious recombination events that cause *RB1* loss of function. Furthermore, a number of other genes and miRNA targets have also been implicated in the progression and severity of RB and other cancers. Further complexity emerges from the network of genes, miRNAs, and other small RNAs whose functions modify *RB1* mRNA abundance and *RB1* protein availability. These include not only protein-coding genes in the immediate vicinity of *RB1* but also miRNAs and other genes located elsewhere in the genome. Altogether, we conclude that *RB1* loss of function represents the tip of an iceberg of events that determine RB development, progression, severity, and disease risk. Comprehensive assessment of personalized RB risk will require genetic and epigenetic evaluations beyond *RB1* protein coding sequences.

### Conflict of interest statement

The authors declare that the research was conducted in the absence of any commercial or financial relationships that could be construed as a potential conflict of interest.
